# Hydrogen Peroxide Triggers a Dual Signaling Axis To Selectively Suppress Activated Human T Lymphocyte Migration

**DOI:** 10.4049/jimmunol.1600868

**Published:** 2017-03-31

**Authors:** Jennifer A. Ball, Isabella Vlisidou, Matthew D. Blunt, Will Wood, Stephen G. Ward

**Affiliations:** * Department of Pharmacy and Pharmacology, University of Bath, Bath BA2 7AY, United Kingdom; and; † Department of Cellular and Molecular Medicine, Faculty of Biomedical Sciences, University of Bristol, Bristol BS8 1TD, United Kingdom

## Abstract

H_2_O_2_ is an early danger cue required for innate immune cell recruitment to wounds. To date, little is known about whether H_2_O_2_ is required for the migration of human adaptive immune cells to sites of inflammation. However, oxidative stress is known to impair T cell activity, induce actin stiffness, and inhibit cell polarization. In this study, we show that low oxidative concentrations of H_2_O_2_ also impede chemokinesis and chemotaxis of previously activated human T cells to CXCL11, but not CXCL10 or CXCL12. We show that this deficiency in migration is due to a reduction in inflammatory chemokine receptor CXCR3 surface expression and cellular activation of lipid phosphatase SHIP-1. We demonstrate that H_2_O_2_ acts through an Src kinase to activate a negative regulator of PI3K signaling, SHIP-1 via phosphorylation, providing a molecular mechanism for H_2_O_2_-induced chemotaxis deficiency. We hypothesize that although H_2_O_2_ serves as an early recruitment trigger for innate immune cells, it appears to operate as an inhibitor of T lymphocyte immune adaptive responses that are not required until later in the repair process.

## Introduction

Reactive oxygen species (ROS) are known to influence the outcome of T cell responses. Depending on concentration, exposure time, and microenvironment, the effects of ROS on T cells can be very distinct and affect a variety of physiological events, including cell proliferation, host defense, differentiation, apoptosis, senescence, and activation of growth-related signaling pathways. T cells can physiologically produce low levels of H_2_O_2_ upon TCR and chemokine stimulation, which have been shown to facilitate T cell activation (1, 2). Additionally, T lymphocytes are known to express NADPH oxidase enzymes NOX2 (2) and DUOX1 that catalyze the reduction of molecular oxygen to generate superoxide O_2_^−^, which can dismute to generate ROS species. These ROS participate in host defense by killing or damaging invading microbes (3). However, in several human pathologies, including cancer and a variety of autoimmune disorders, high levels of pro-oxidants are known to induce T lymphocyte hyporesponsiveness (4). In cancer, this can be harmful due to suppression of potentially tumor-reactive T cells (5), whereas in autoimmune disease high levels of ROS are thought to help control self-reactive T cells. As such, the level of ROS within the microenvironment appears to be an important control mechanism influencing T cell fate.

H_2_O_2_ has been demonstrated to act as an important early damage cue triggering innate immune cell migration in *Drosophila* and zebrafish models of in vivo inflammation (6–8). Additionally, H_2_O_2_ has been shown to act as a chemoattractant for mouse peritoneal neutrophils at low concentrations (9), and human neutrophil chemotaxis in response to gradients of H_2_O_2_ has been observed in vitro (8). Thus, H_2_O_2_ appears to be required for innate immune cell migration; however, little is known about whether H_2_O_2_ is required for the migration of human adaptive immune cells. Recently, H_2_O_2_ uptake, through aquaporin-3, was shown to be required for efficient mouse T lymphocyte migration toward CXCL12 (1), suggesting a role in regulating the migration of adaptive immune cells.

Unraveling the mechanism through which H_2_O_2_ modulates signaling pathways is vital for understanding its role in T lymphocyte biology. It is widely accepted that H_2_O_2_ and ROS can act as second messengers through their ability to reversibly oxidize specific cysteine residues in proteins (10). Indeed, ROS can oxidize phosphatases (11), kinases (8) transcription factors (12), and ion channels (13) to alter intracellular signaling. Cellular signaling cascades commonly activated by several types of ROS include the PI3K pathway and Src family kinases (SFKs), which regulate cellular survival activation and migration, thus establishing a link between oxidative conditions and cellular signaling (14, 15). H_2_O_2_ has been shown to enhance PI3K signaling by inactivating the lipid phosphatase PTEN (14). PI3K can also be negatively regulated by the SHIP-1 that is primarily expressed in hematopoietic cells. SHIP-1 dephosphorylates phosphatidylinositol (3,4,5)-trisphosphate [PtdIns(3,4,5)P_3_], generating phosphatidylinositol (3,4)-bisphosphate, which leads to inhibition of pleckstrin homology domain–containing enzymes, that are dependent on PtdIns(3,4,5)P_3_ for their activation. Whether H_2_O_2_ has an effect on SHIP-1 has yet to be determined.

In this study, we show that oxidative signaling inhibits T lymphocyte chemotaxis to the inflammatory chemokine CXCL11, without affecting migration to CXCL12 or CXCL10. We go on to show that this H_2_O_2_-induced chemotactic deficiency is due to both reduced surface expression of CXCR3 as well as SHIP-1 activation through activation of a redox-sensitive SFK. Similarly, pharmacological activation of SHIP-1 with the allosteric activator AQX1 impaired CXCL11-induced chemotaxis by manipulating PI3K signaling and ezrin, radixin, and moesin (ERM) phosphorylation, providing an exciting new mechanism for the targeted inhibition of PI3K-mediated signaling in leukocytes with potential therapeutic opportunities in T lymphocyte–driven pathologies.

## Materials and Methods

### Chemicals

PP2 is an ATP competitive inhibitor of SFKs purchased from Calbiochem. An allosteric SHIP-1 activator referred to as AQX1 was supplied to S.G.W. by Aquinox for research purposes only, the structure of which is shown in Supplemental Fig. 1A.

### PBMC and naive CD4^+^ T lymphocyte isolation from whole blood

Blood (50–100 ml) was collected aseptically from healthy volunteers of both sexes within an age range of 21–65 y into a sterile disposable 50-ml syringe prepared with 2 U/ml heparin (Sigma-Aldrich). The blood was subsequently diluted with warm sterile RPMI 1640 medium without supplements in a ratio of 1:1. Of the diluted blood, 25–35 ml was carefully layered onto 15 ml of Lymphoprep (Axis-Shield) in 50-ml plastic tubes. Preparations were spun at 250 × *g* for 30 min; following centrifugation, the mononuclear cell–rich interface containing the PBMCs was diluted 1:1 with RPMI 1640 medium. PBMCs were washed by centrifugation and resuspended in unsupplemented RPMI 1640 medium. Naive CD4^+^ T lymphocytes were isolated from the freshly isolated PBMCs using the naive CD4^+^ T cell isolation kit II (Miltenyi Biotec) and an LS column following the manufacturer’s instructions.

To clonally expand CD4^+^ lymphocytes, the washed PBMCs were resuspended in fully supplemented RPMI 1640 medium (10% FCS, 10 μg/ml penicillin, 10 μg/ml streptomycin) at an equal volume to that of the original volume of whole blood collected from the donor. Staphylococcal enterotoxin B (SEB) (Sigma-Aldrich) was added at a final concentration of 1 μg/ml for 72 h. The suspension cells were then washed in unsupplemented RPMI 1640 medium and resuspended in the same volume of complete medium supplemented with IL-2 (Chemicon) at a final concentration 36 U/ml. The cells were washed, resuspended, and supplemented with fresh IL-2 every 2 d for 10–14 d to maintain cell confluency between 0.1 and 1 × 10^6^ cells/ml. Cells were used 8–14 d after isolation and rested in complete medium without IL-2 for 16 h before use. All procedures using human blood were carried out under the University of Bath safety and ethical guidelines for the use of human tissue.

### Flow cytometry

Freshly isolated naive CD4^+^ and SEB-activated T lymphocytes were washed three times by centrifugation at 250 × *g* and resuspended in nonsupplemented RPMI 1640 medium. After treatment with vehicle or activator as described in the figure legends, cells were fixed using BD fixation and permeabilization solution (containing formaldehyde; BD Biosciences) for 30 min at 4°C to allow intracellular staining of proteins. Cells were washed twice in BD Perm/Wash solution (containing sodium azide and saponin; BD Biosciences) solution and incubated with 0.02% (v/v) anti–phospho-ERM (Cell Signaling Technology) and anti–phospho-SHIP-1 (Cell Signaling Technology) diluted in BD Perm/Wash for 30 min at 4°C. Cells were washed twice by centrifugation at 250 × *g* and resuspension in BD Perm/Wash solution and then incubated with 0.01% (v/v) anti-rabbit IgG FITC-conjugated secondary Ab (Sigma-Aldrich) for 30 min at 4°C. Alternatively, cells were stained with tetramethylrhodamine isothiocyanate (TRITC)–conjugated phalloidin for 30 min. Cells were washed twice in BD Perm/Wash solution and then resuspended in 400 μl of ice-cold PBS plus 1% (v/v) FBS in 5-ml rounded-bottom FACS tubes for analysis by a BD FACSCanto II flow cytometer and BD FACSDiva software (BD Biosciences). TRITC-stained cells were excited at 547 nm and emission was recorded at 573 nm, whereas FITC-stained cells were excited at 495 nm and emission was recorded at 519 nm. Mean fluorescence intensity was used to describe the level of phosphorylated ERM, SHIP-1 protein, or actin polymerization.

### Surface receptor expression by flow cytometry

SEB-activated T lymphocytes were resuspended in fully supplemented RMPI 1640 medium at a concentration of 1 × 10^6^ cells/ml. Cells were treated with either vehicle or stated concentrations of drugs for 30 min. Cells were washed twice with ice-cold FACS buffer (PBS plus 2% FCS) and then resuspended in diluted 1:50 (v/v) PE-conjugated anti-CXCR3 Ab (R&D Systems) or PE-conjugated IgG isotype control (1:50 [v/v] dilution; R&D Systems). Alternatively, cells were labeled with either anti-human CD11a PE-conjugated mAb (1:50 [v/v] dilution; ImmunoTools), anti-human CD49d PE-conjugated mAb (ImmunoTools), or PE-conjugated IgG Ab. Cells were labeled on ice for 1 h and then washed twice more in FACS buffer. Alternatively, cells were incubated with primary Ab, that is, 10 μg/ml KIM127 mAb (gift from N. Hogg), for 30 min and then washed and incubated with secondary anti-mouse FITC-conjugated Ab (dilution 1:50 [v/v]). Alternatively, cells were incubated with Live/Dead stain Zombie Yellow and CD3 PerCP–, CD4 Alexa Fluor 700–, CD8 PE-Cy7–, CD127 PE-Cy5–, and CD25 Brilliant Violet 421–conjugated mAbs (BioLegend) for 30 min on ice. Finally, cells were resuspended in PBS and placed in 5-ml round-bottom tubes for analysis by flow cytometry. PE fluorescence was detected by exciting cells at 496 nm, and emission was recorded at 576 nm by a BD FACSAria flow cytometer and analyzed by BD FACSDiva software. Mean fluorescence intensity was used to describe the level of CXCR3, CD11a, and CD49d expression, and percentage positive was used to describe the subsets of T lymphocytes.

### Chemotaxis assay

Cell migration assays were performed using a 96-well plate-based chemotaxis system (Neuro Probe). Cells were suspended in phenol red–free RPMI 1640 medium with 0.1% BSA at 3.2 × 10^6^ cells/ml and treated as described in each figure legend. Lower chambers of the 96-well plate were loaded with 29 μl of RPMI 1640 medium (basal conditions) or CXCL11 (10 nM; PeproTech). This was overlaid with a 5-μm pore-size filter, and 25 μl of the cell suspension was placed above each required well. The plate was incubated at 37°C, 5% CO_2_ for 3 h, after which viable cells in the bottom chamber were counted by flow cytometric analysis. Cell viability was routinely determined with annexin V and propidum iodide staining followed by flow cytometry.

### SHIP-1 immunoprecipitation and malachite green phosphate assay

The malachite green phosphate assay was carried out using full-length rSHIP-1 (provided by GlaxoSmithKline) and immunoprecipitated SHIP-1. SEB-activated T lymphocytes were collected by centrifugation at 250 × *g* and resuspended in serum-free RMPI 1640 medium. Cells (1 × 10^6^) were lysed for each reaction of the malachite green assay. Samples were rotated for 30 min at 4°C to assist cell lysis, after which cells were centrifuged at 153 × *g*, 4°C for 10 min. The supernatant containing SHIP-1 was incubated overnight in 0.01% (v/v) SHIP-1 mAb (Cell Signaling Technology). A/G agarose beads (PeproTech) (5 μl per point of malachite green) were washed twice in lysis buffer without inhibitors, resuspended in 100 μl, and added to the cell lysis of the SEB-activated T cells for at least 2 h. Samples were then washed three times in 1 ml of lysis buffer and three times in malachite green reaction buffer (5% [v/v] glycerol, 20 mM Tris HCl, 10 mM MgCl_2_ [pH 7.4]) to remove all proteins not attached to the A/G agarose beads and resuspended in 20 μl of reaction buffer per point. The immunoprecipitated SHIP-1 was then used in the malachite green phosphate assay. Full-length rSHIP-1 was diluted to 1 μM in reaction buffer and assessed in the malachite green reaction. SHIP-1 can dephosphorylate the in vitro substrate d-inositol(1,3,4,5) tetrakisphosphate to produce inositol-1,3,4-trisphosphate and free phosphate. A malachite green solution can react with the free phosphate to give a rapid color change from yellow to green. Twenty microliters of immunoprecipitated SHIP-1 solution or 1 μM recombinant SHIP-1 was added to each of the required wells of a 96-well plate and incubated with SHIP-1 activators, inhibitors, or H_2_O_2_ at given concentrations for 5 min at room temperature. Substrate d-inositol(1,3,4,5) tetrakisphosphate (100 μM) (Echelon Biosciences) was added for 30 min at 37°C. One hundred microliters of malachite green solution (Echelon Biosciences) was added to each well and incubated for 15 min at room temperature in the dark. The plate was then read at 650 nm using a FLUOstar Optima plate reader.

### Immunoblotting

To analyze the effect of exogenous H_2_O_2_ on SHIP-1 activity, cells were treated as described in RPMI 1640 medium, centrifuged, and lysed in 50 μl of lysis buffer (50 mM Tris-HCl [pH 7.5], 150 mM NaCl, 1% Nonidet P-40, 5 mM EDTA, 1 mM sodium vanadate, 1 mM sodium molybdate, 10 mM sodium fluoride, 40 μg/ml PMSF, 0.7 μg/ml pepstatin A, 10 μg/ml aprotinin, 10 μg/ml leupeptin, and 10 μg/ml soybean trypsin inhibitor). Samples were rotated at 4°C for 20 min and centrifuged at 600 × *g* for 10 min. Supernatant was transferred to fresh tubes and diluted 1:5 with 10% SDS containing sample buffer, and samples were subjected to SDS-PAGE electrophoresis and immunoblotting following standard procedures.

### Adhesion assay

A flat-bottom 96-well plate was coated with either 10 μg/ml recombinant human fibronectin (R&D Systems) or 10 μg/ml recombinant human ICAM-1 (R&D Systems) overnight. The plate was washed twice with prewarmed PBS, then unsupplemented RPMI 1640 medium (without phenol red). SEB-activated cells were washed twice into unsupplemented medium and treated with the stated concentrations of compounds for 30 min. Cells were then added to the 96-well plate and stimulated with the TCR antagonist UCHT1 at a concentration of 10 μg/ml. The plate was sealed and incubated for 30 min at 37°C to allow cells to adhere. The plate was then inverted for 15 min, after which the seal was removed while the plate was still inverted, all medium and nonadherent cells were removed from plate, and wells were washed gently with PBS. The adherent cells were quantified using the MTT assay as per the manufacturer’s instructions, and absorbance was recorded using a FLUOstar Optima plate reader at 540 nm.

### Immunostaining and confocal microscopy

Cells (1 × 10^6^) were stimulated as required and fixed in BD fixation and permeabilization solution (BD Biosciences) for 30 min at 4°C. Cells were washed twice via centrifugation at 250 × *g* and resuspension in BD Perm/Wash solution (containing sodium azide and saponin; BD Biosciences) and then incubated with 0.02% (v/v) anti–phospho-ERM Ab (Cell Signaling Technology) in BD Perm/Wash solution overnight at 4°C. Cells were washed twice in BD Perm/Wash solution and resuspended in 0.01% (v/v) goat anti-rabbit FITC-conjugated secondary Ab diluted in Perm/Wash solution overnight at 4°C. Cells were then washed and resuspended in 20 μl of permeabilization solution, and cells were pipetted into Mowiol to adhere coverslips to slides. Cells were visualized using a Zeiss LSM 510 Meta confocal microscope using Plan-Apochromat ×63/1.4 oil differential interference contrast objectives. Cells were excited by 488 nm light, and fluorescence emission was collected at 520 nm with a band-pass filter of 530 ± 15 nm. Images were collected at ×2 zoom.

### Statistical analysis

Data were normalized as described in the figure legends. Values are presented as mean ± SEM, and *n* represents the total number of donors or individual repeats of each study. Statistical analysis was undertaken using GraphPad Prism software. Data were analyzed using a one-way ANOVA followed by a Dunnett posttest to determine significant difference as compared with control, or when there were two independent variables a two-way ANOVA with a Bonferrori posttest was used. A *p* value <0.05 was considered significant.

## Results

### H_2_O_2_ potently impairs T lymphocyte migration to CXCL11 but not to CXCL12 or CXCL10

**FIGURE 1.**
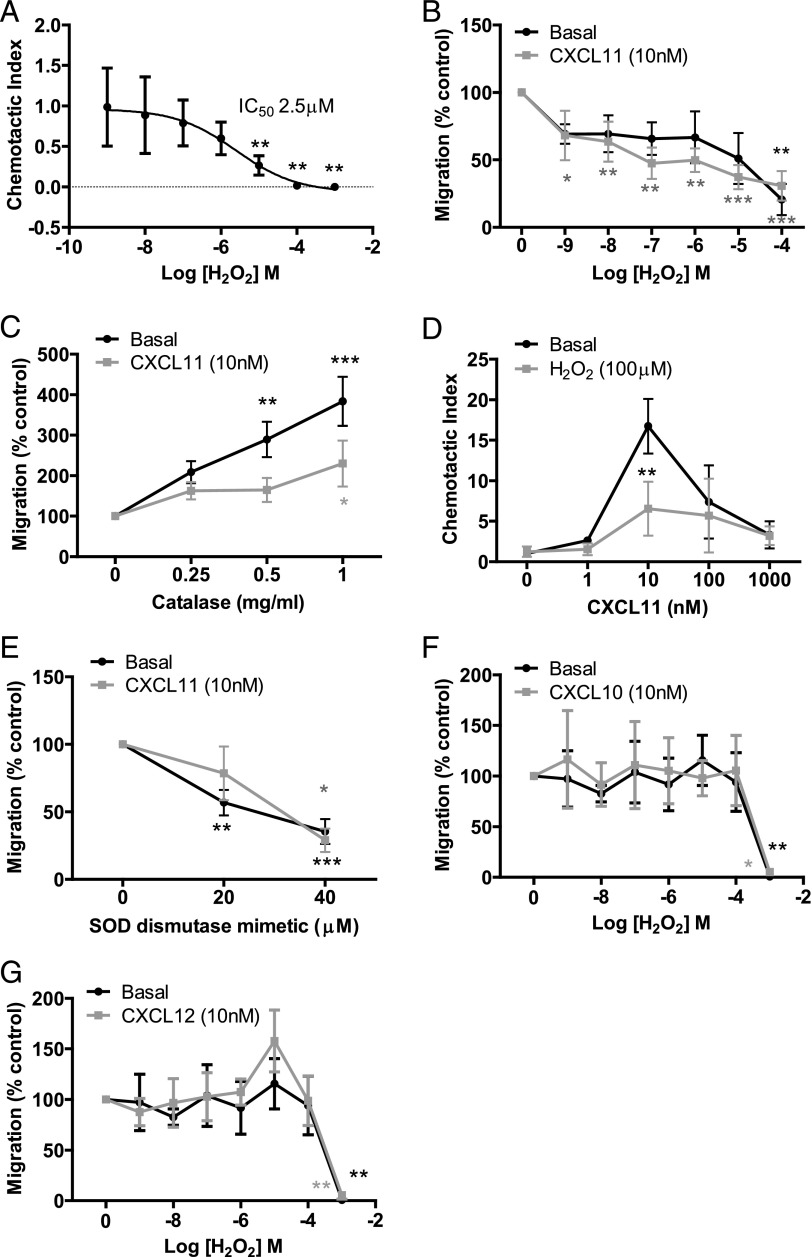
H_2_O_2_ potently inhibits T lymphocyte migration to CXCL11. (**A**) SEB-activated T cell migration toward either basal or increasing concentrations of H_2_O_2_. **p < 0.01. (**B**) Basal or CXCL11 (10 nM)-induced migration of SEB-activated T cells pretreated with increasing concentrations of H_2_O_2_. *p < 0.05, **p < 0.01, ***p < 0.001. (**C**) Basal or chemokine-induced migration of SEB-activated T cells pretreated with indicated concentrations of catalase. *p < 0.05, ^##^*p* < 0.01, ^###^*p* < 0.001. (**D**) SEB-activated T cells pretreated with H_2_O_2_ (100 μM) migration toward increasing concentrations of CXCL11. *p < 0.05, **p < 0.01, ***p < 0.001. (**E**) Basal or CXCL11 (10 nM)-induced migration of SEB-activated T cells pretreated with increasing concentrations of the superoxide dismutase mimetic Mn(III)tetrakis(4-benzoic acid)porphyrin chloride. *p < 0.05, **p < 0.01, ***p < 0.001. (**F**) Basal or CXCL10 (10 nM)-induced migration of SEB-activated T cells pretreated with increasing concentrations of H_2_O_2_. *p < 0.05, **p < 0.01. (**G**) Basal or CXCL12 (10 nM)-induced migration of SEB-activated T cells pretreated with increasing concentrations of H_2_O_2_. *p < 0.05, **p < 0.01. For all graphs, migrated cells were counted using flow cytometry; data are the mean values ± SEM of three or four independent experiments.

To investigate the effect of H_2_O_2_ on migration of human T lymphocytes, we used a neuroprobe migration assay to first test whether H_2_O_2_ can act as a chemoattractant for SEB-activated human T cells. Surprisingly, H_2_O_2_ added to the bottom well of the neuroprobe plate caused a concentration-dependent decrease in SEB-activated T lymphocyte migration when compared with media alone_._ Migration was significantly impaired at 10 μM H_2_O_2_, and by 1 mM it was almost completely inhibited, with an IC_50_ of 2.5 μM (Fig. 1A).

Because H_2_O_2_ appears to operate as a negative regulator of T cell migration, we investigated the effect of pretreating T lymphocytes directly with H_2_O_2_ to further examine its impact on both basal and CXCL11-induced migration. Basal migration was not significantly inhibited except at a concentration of 100 μM H_2_O_2_ (Fig. 1B). However, CXCL11-induced SEB-activated T lymphocyte migration was significantly reduced by H_2_O_2_ in a concentration-dependent manner (Fig. 1B). Consistent with this negative role of H_2_O_2_, scavenging of extracellular H_2_O_2_ via the addition of catalase resulted in an increase of both basal and CXCL11-stimulated T cell migration (Fig. 1C), whereas heat-inactivated catalase had no effect (data not shown).

We next investigated the chemotactic responses of SEB-activated T lymphocytes toward increasing concentrations of CXCL11 following pretreatment with 100 μM H_2_O_2_. Maximal migration occurred at 10 nM for both vehicle control and H_2_O_2_-pretreated cells, demonstrating that H_2_O_2_ does not alter T lymphocyte sensitivity to CXCL11 (Fig. 1D). Maximal migration could not be reached by increasing the concentration of CXCL11, suggesting that H_2_O_2_ is not acting as a competitive inhibitor of CXCL11. Consistent with H_2_O_2_ pretreatment, pretreatment with the superoxide dismutase mimetic Mn(III)tetrakis(4-benzoic acid)porphyrin chloride (which, like H_2_O_2_ pretreatment, increases intracellular ROS, as shown in Supplemental Fig. 2) inhibited SEB-activated T lymphocyte basal and CXCL11-induced migration (Fig. 1E).

As H_2_O_2_ significantly impaired migration toward CXCL11, we next investigated its effect on chemotactic migration to another CXCR3 ligand, the inflammatory chemokine CXCL10, as well as the homeostatic chemokine CXCL12 that binds to CXCR4 (Fig. 1F, 1G). Although CXCL11 and CXCL10 are both induced by IFN-γ and thought to promote Th1 immune responses (16–18), they have previously been shown to have distinct expression patterns, potencies, and efficacies in a number of assays, including internalization and migration (19–21). In the neuroprobe assay, CXCL11 significantly increased SEB T lymphocyte migration 6.9 ± 0.9-fold over basal migration, whereas CXCL10 was less potent and migration was increased 3.1 ± 0.9-fold over basal migration (data not shown). That CXCL10 and CXCL11 exhibit distinct efficacies to CXCR3 suggests that they interact with CXCR3 in different ways and are likely to stabilize different conformations of the receptor (22). Strikingly, H_2_O_2_ exposure had no effect on CXCL10-induced migration, with migration only becoming inhibited upon exposure to extremely high concentrations (1 mM) (Fig. 1F). This shows that H_2_O_2_ specifically affects CXCL11-induced migration and not migration to CXCL10 despite the two engaging the same receptor (CXCR3).

### H_2_O_2_ causes a defect in actin polarization in T cells but has no effect on adhesion

**FIGURE 2.**
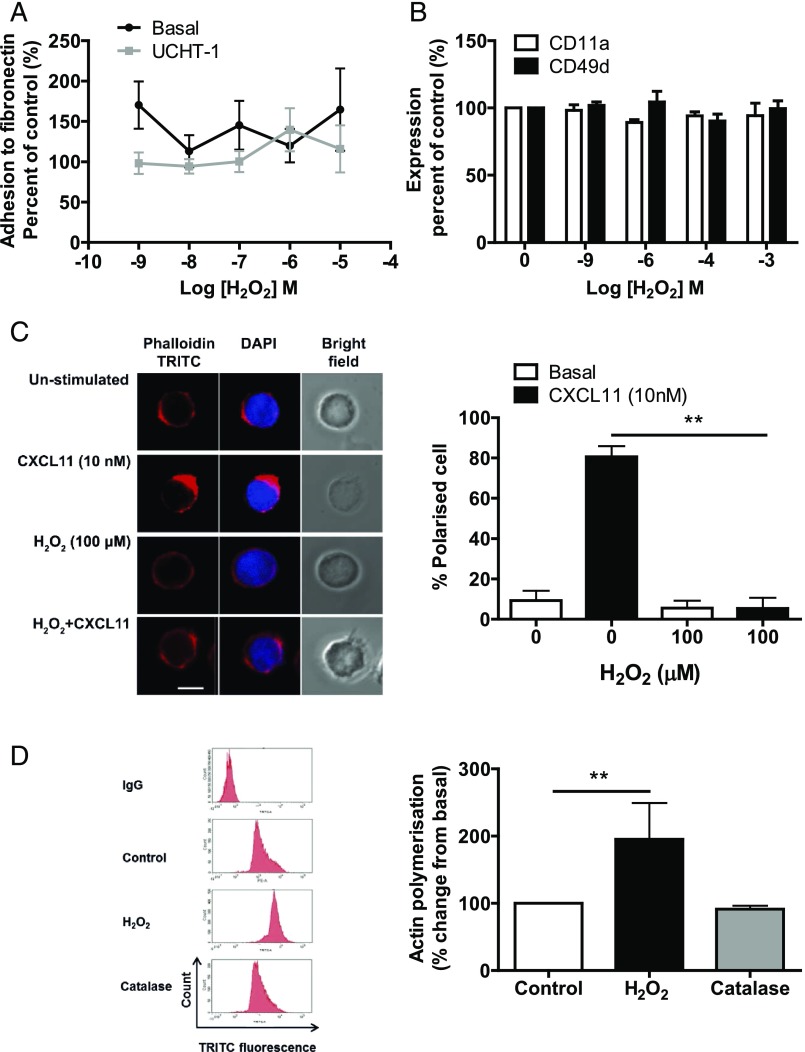
Effect of H_2_O_2_ and CXCL11 on polarization of SEB-activated T lymphocytes. (**A**) SEB-activated T lymphocytes (1 × 10^6^) were pretreated with increasing concentrations of H_2_O_2_ for 30 min. Cells were stimulated with UCHT1 (10 μg/ml) for 5 min, before being allowed to adhere to fibronectin (10 μg/ml)-coated 96-well plates for 30 min. The plate was sealed and turned upside down for 15 min. Wells were washed gently with PBS to remove any unadhered cells. Cells were then scraped and counted using flow cytometry. Graph shows percentage change from basal. (**B**) Cells were treated with increasing concentrations of H_2_O_2_ for 30 min and then the expression of CD11a and CD49d was assessed using PE-conjugated Abs and analyzed by flow cytometry. Data are represented as percentage change from basal and mean ± SEM of three independent experiments. (**C**) SEB-activated T lymphocytes (1 × 10^6^; 8–12 d after isolation) were pretreated with 100 μM H_2_O_2_ for 30 min and then stimulated with 10 nM CXCL11 for 5 min. Then, cells were fixed in BD fixation reagent, permeabilized with BD permeabilization reagent, and stained with TRITC-tagged phalloidin and DAPI. The left panel shows immunofluorescence microscopy representative images showing actin and DAPI staining. Scale bar, 10 μm. The right panel represents the mean ± SEM percentage of polarized cells observed from three independent donors, with 20 cells counted for each condition. Statistical significance was determined by two-way ANOVA with a Bonferroni posttest. **p < 0.01 compared with control. (**D**) H_2_O_2_, but not catalase, increases actin polymerization of SEB-activated T lymphocytes. One million SEB-activated T lymphocytes (8–12 d after isolation) were washed three times in serum-free medium and treated with H_2_O_2_ (100 μM) or catalase (1 mg/ml) for 30 min. Cells were fixed using BD fixation reagent for 20 min, permeabilized, and incubated with TRITC-tagged phalloidin for 30 min. Mean fluorescence intensity per 10,000 cells was measured using flow cytometry. Data are (left panel) representative FAC plots from one donor and (right panel) mean ± SEM minus IgG control, normalized to the untreated control from three independent donors. Statistical significance was determined by a one-way ANOVA with a Dunnett posttest. **p < 0.01 compared with control.

Ligation of the TCR is known to increase adhesion of T lymphocytes to both the extracellular matrix and other cells, which is critical for their migration, extravasation, and formation of immunological synapses (23). The adhesion of SEB-activated T lymphocytes was examined following H_2_O_2_ treatment to determine whether altered adhesion could be the underlying reason for their reduced ability to migrate. Interestingly, H_2_O_2_ had no effect on basal or UCHT1-induced adhesion to fibronectin (Fig. 2A) and had no effect on the expression of the α-subunit of integrin receptors LFA-1 (CD11a subunit) or α_4_β_1_ (CD49 subunit) (Fig. 2B). H_2_O_2_-induced inhibition of T cell chemotaxis is therefore not due to disrupted adhesion.

In response to chemotactic signals, T cells reorganize their actin cytoskeleton and become polarized toward the chemoattractant gradient, which leads to chemotaxis and T cell trafficking (24, 25). CXCL11-stimulated T cells develop a polarized morphology at the leading edge known as the uropod (26), which was visualized with phalloidin staining (Fig. 2C). In contrast, H_2_O_2_-treated T cells failed to develop uropods; instead, they exhibited irregular patch-like F-actin staining (Fig. 2D). CXCL11-induced polarization was significantly impaired in H_2_O_2_-treated T cells (Fig. 2C). Interestingly, H_2_O_2_ significantly increased the level of polymerized F-actin detected to 195 ± 30% of control levels, with catalase pretreatment being able to rescue the effect on the level of polymerized F-actin (Fig. 2D). Our data suggest that high concentrations of H_2_O_2_ could impair T lymphocyte migration by misregulation of the actin cytoskeleton inhibiting the ability of the cell to polarize toward CXCL11.

### SFKs are required for the H_2_O_2_-induced decrease in CXCR3 surface expression

**FIGURE 3.**
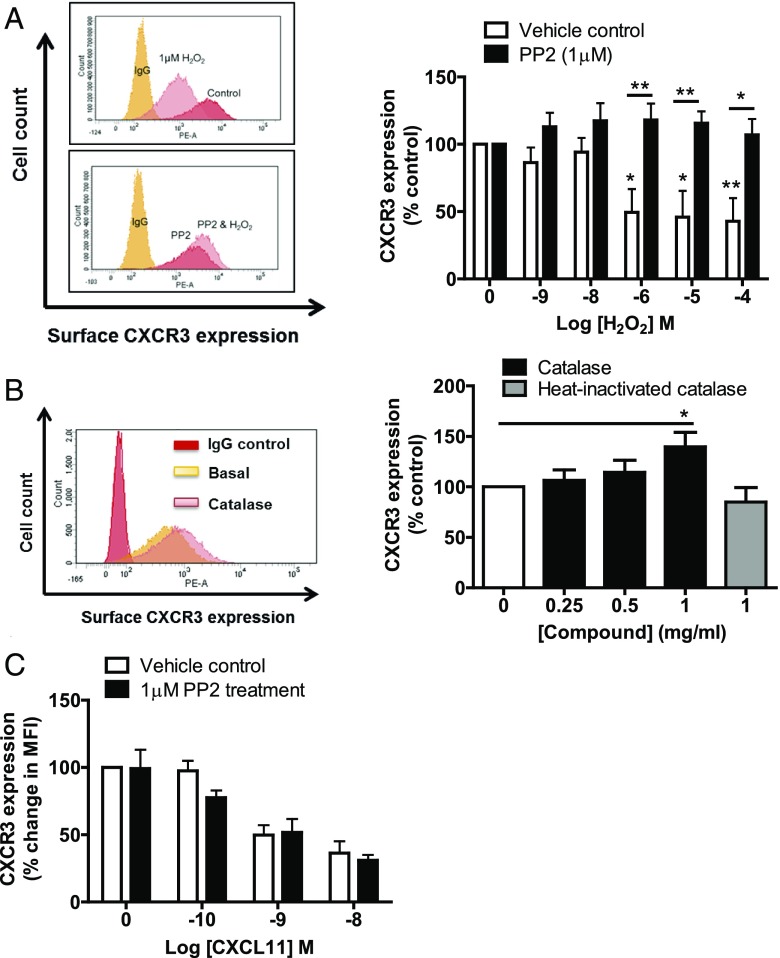
Exogenous H_2_O_2_ requires Src kinase to reduce surface expression of CXCR3. (**A**) The left panel shows representative FACS plots from a single donor showing the effect of H_2_O_2_ on CXCR3 expression in SEB-activated T lymphocytes with or without PP2 (1 μM) treatment. The right panel shows the mean ± SEM of three independent donors, normalized to the untreated control. Statistical significance was determined by a two-way ANOVA with a Bonferroni posttest. *p < 0.05, **p < 0.01. (**B**) The left panel shows a representative FACS plot for a single donor showing the effect of catalase on CXCR3 expression. The right panel shows the mean ± SEM of three independent donors, normalized to the untreated control. Statistical significance was determined by a one-way ANOVA with a Dunnett posttest. *p < 0.05, as compared with control. (**C**) Graph showing effect of increasing concentrations of CXCL11 on CXCR3 expression in SEB-activated T lymphocytes pretreated with Src kinase inhibitor PP2 (1 μM). Level of surface CXCR3 expression was determined using PE-conjugated CXCR3 Ab. Data are mean values ± SEM of three independent experiments.

One potential mechanism by which H_2_O_2_ may act as an inhibitor of CXCL11-induced migration and actin polarization is by affecting the trafficking of the CXCL11 receptor. Following Ag encounter, the chemokine receptor CXCR3 is upregulated on T lymphocytes and is crucial for their CXCL11-guided recruitment into inflamed tissues (17). To determine the effect of H_2_O_2_ on CXCR3 surface expression, SEB-activated T lymphocytes were treated with increasing concentrations of H_2_O_2_ for 30 min and CXCR3 surface expression was determined by flow cytometry. H_2_O_2_ treatment caused a reduction in CXCR3 expression with an IC_50_ of 2 μM (Fig. 3A). Thus, H_2_O_2_ significantly decreased the expression of CXCR3 at concentrations that affect migration but are not cytotoxic (Supplemental Fig. 3) and have no effect on polarization to different T cell subsets (Supplemental Fig. 4A–C). Because cells were exposed to H_2_O_2_ for only 30 min prior to analysis, this change in surface expression is unlikely to be due to transcriptional changes, but is more likely due to an effect on CXCR3 trafficking. Consistent with this, we found that addition of catalase led to an increase in surface expression levels of CXCR3, which was not observed when using heat-inactivated catalase (Fig. 3B).

Studies in zebrafish and *Drosophila* have identified the SFK Lyn (Src42A in *Drosophila*) as being a target of H_2_O_2_ in blood cells (8, 27), with this kinase containing a critical redox-sensitive cysteine residue. To test whether Src family kinases are playing a role in H_2_O_2_-induced inhibition of T cell migration, cells were pretreated with the SFK inhibitor PP2, which abrogated downregulation of CXCR3 surface expression triggered by H_2_O_2_ (Fig. 3A). This suggests, therefore, that H_2_O_2_-induced CXCR3 downregulation in activated T lymphocytes requires SFK signaling.

CXCR3 internalization occurs rapidly upon agonist activation of the receptor and is a critical mechanism by which G protein–coupled receptor signaling is regulated. As H_2_O_2_ also appears able to regulate the expression of CXCR3 through SFK signaling, we were interested whether CXCL11 also internalizes the receptor through SFK signaling. CXCL11 evoked a concentration-dependent reduction in the expression of CXCR3; however, SFK inhibition had no effect on CXCL11-induced downregulation of CXCR3 (Fig. 3C). This demonstrates that H_2_O_2_ and CXCL11 induce distinct intracellular signaling mechanisms to internalize the CXCR3 receptor.

Our results show that H_2_O_2_ specifically affects CXCL11-induced migration but not migration to CXCL10, despite both engaging the same receptor. We therefore wanted to determine whether they have differential effects on CXCR3 internalization, which could provide a potential explanation for the distinct migratory effects. We and others have previously shown that CXCL11 was more potent than CXCL10 at reducing the expression of CXCR3 (28, 29). Thus, decreased CXCR3 surface expression following H_2_O_2_ exposure could lead to desensitization of the T cell to CXCL11 while leaving sufficient CXCR3 expression for sensitivity toward CXCL10. As expected, CXCL12 triggered internalization of its receptor CXCR4 but not CXCR3 (data not shown). Consistent with its lack of effect on CXCL12-induced migration, H_2_O_2_ had no effect on the surface expression of CXCR4 (data not shown).

### Src kinases are required for H_2_O_2_-induced SHIP-1 phosphorylation and activation of its catalytic activity

**FIGURE 4.**
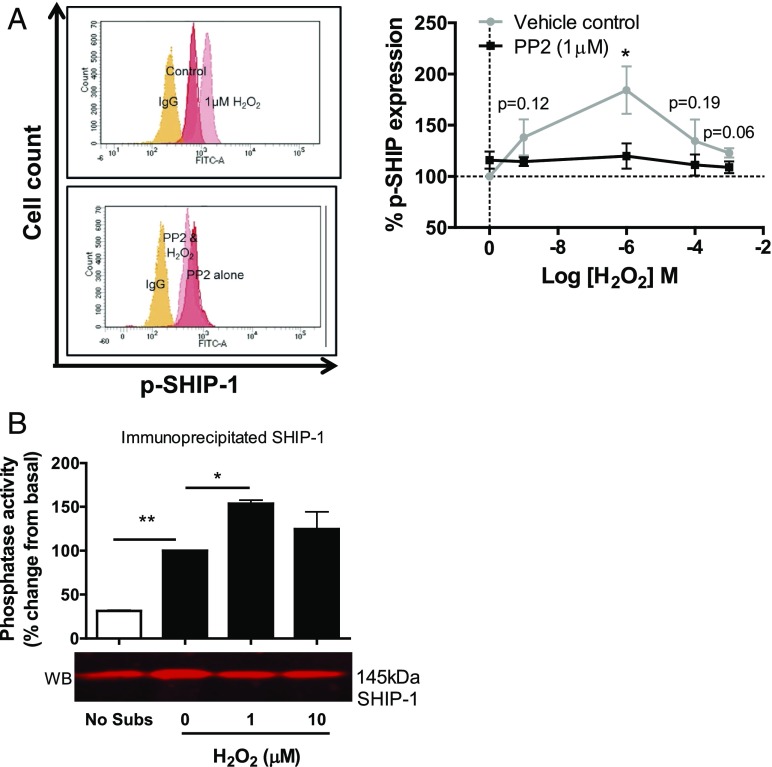
H_2_O_2_ requires an SFK to phosphorylate and enhance the catalytic activity of SHIP-1. (**A**) The left panel shows a representative FACS overlay from one donor showing the effect of H_2_O_2_ on anti–phospho-SHIP-1 levels in T cells with or without PP2 treatment (1 μM). The right panel shows the mean values ± SEM from four independent donors. (**B**) Effect of H_2_O_2_ (1 μM) on the catalytic activity of SHIP-1 protein immunoprecipitated from 1 × 10^6^ SEB-activated T lymphocytes. Catalytic activity was quantified using a malachite green phosphatase assay, and protein concentration was determined by Western blot. Data are mean values ± SEM of three independent experiments. *p < 0.05, **p < 0.01.

The effect of H_2_O_2_ on SHIP-1 has not been established, although SHIP-1 expression has been shown to enhance cell survival in response to oxidative stress (30), and SHIP-1 has been shown to have vital roles in the regulation of human SEB-activated T lymphocytes (31). Upon cell stimulation, SHIP-1 is recruited to the membrane, where it is tyrosine phosphorylated on Y1020, which lies in the second NPXY motif toward its C-terminal. Phosphorylation is used as a marker of activated SHIP-1 when SHIP-1 is located at the cell membrane (32) and is able to bind the phosphotyrosine-binding domains of Shc (33), Dok1, and Dok2 (34). We found that H_2_O_2_ increased SHIP-1 phosphorylation in a concentration-dependent manner; with maximal phosphorylation at 1 μM (Fig. 4A), SHIP-1 phosphorylation occurs at concentrations of H_2_O_2_ that do not affect cellular viability, but do significantly alter the migration of T lymphocytes to CXCL11. Additionally, we examined the effect of high concentrations H_2_O_2_ on total SHIP-1 protein by Western blot and determined that H_2_O_2_ has no effect on degradation of SHIP-1 (Supplemental Fig. 4D).

Because SHIP-1 phosphorylation has previously been described to be dependent on SFKs in B lymphocytes (35), we wondered whether this H_2_O_2_-mediated effect might also be operating via SFKs. We found that PP2 significantly impaired H_2_O_2_-induced SHIP-1 phosphorylation (Fig. 4A), indicating that H_2_O_2_-induced SHIP-1 phosphorylation does indeed require SFK activity. As SHIP-1 phosphorylation is used as a marker of activated SHIP-1, we wanted to determine whether H_2_O_2_ has an effect on the catalytic ability of SHIP-1. Subsequent analysis showed that H_2_O_2_ had no effect on the catalytic ability of rSHIP-1 on a malachite green phosphatase assay (data not shown). Therefore, unlike PTEN, the catalytic activity of SHIP-1 does not appear to be decreased by oxidation.

Because SHIP-1 phosphorylation required SFK activity, we wanted to understand how H_2_O_2_-induced cellular signaling could alter the catalytic activity of native SHIP-1. Directly treating immunoprecipitated SHIP-1 with H_2_O_2_ does not allow for any H_2_O_2_-induced signaling that could alter the catalytic activity of SHIP-1 within the cell. Therefore, SHIP-1 was immunoprecipitated from either untreated or H_2_O_2_-treated T cells and the catalytic activity was determined by malachite green assay. The amount of immunoprecipitated SHIP-1 in each sample was determined by Western blot. SHIP-1 immunoprecipitated from the cells treated with H_2_O_2_ had significantly increased catalytic activity as compared with the untreated controls (Fig. 4B). These results show that oxidative signaling plays a key role in activating the lipid phosphatase SHIP-1 via an SFK and that this SHIP activation then negatively regulates CXCL11-induced migration.

### Pharmacological activation of SHIP-1 inhibits the migration of lymphocytes by reducing PI3K signaling and modulating ERM protein phosphorylation

**FIGURE 5.**
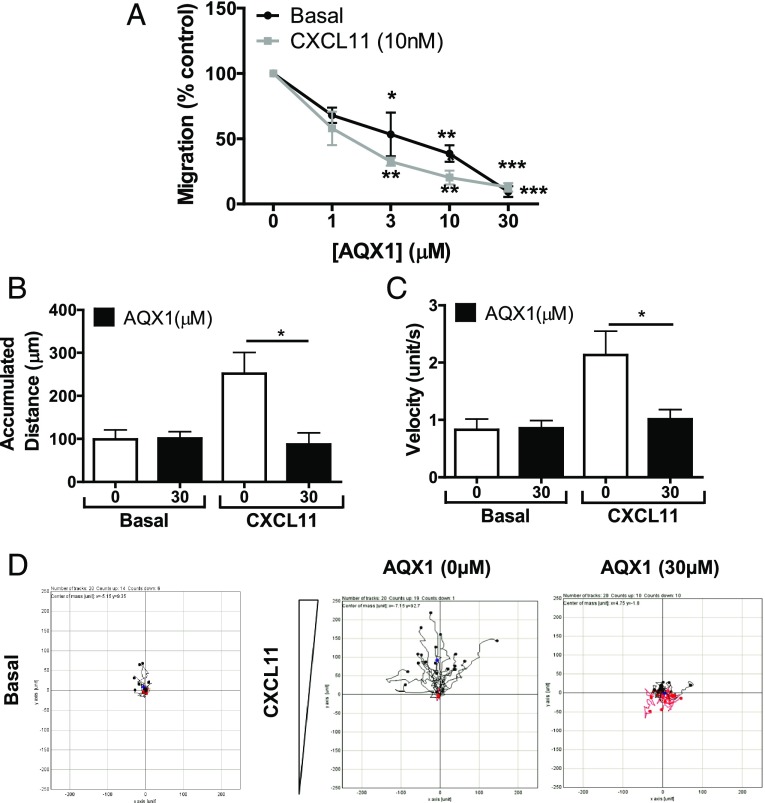
SHIP-1 modulation reduces CXCL11-mediated migratory responses in activated T lymphocytes. (**A**) Previously SEB-activated T cells were treated with AQX1 (1–30 μM) for 30 min. Basal and chemotactic migration to CXCL11 (10 nM) was assessed using the Neuro Probe chemotaxis assay as described in *Materials and Methods*. Data are expressed as percentage inhibition of migration and are means ± SEM of three independent experiments. (**B**–**D**) Additionally, the migration of previously activated T cells treated with AQX1 (30 μM) with or without CXCL11 (100 nM) gradient was also assessed using ibidi two-dimensional chemotaxis μ-slides and video microscopy as described in *Materials and Methods*. Data represent three separate experiments with 20 cells recorded under each condition in each experiment, where (B) accumulated distance (micrometers) and (C) velocity (units/second) are the means ± SEM from three independent experiments. (D) Individual tracks of cell tracks from a single representative experiment. *p < 0.05, **p < 0.01, ***p < 0.001 (one-way ANOVA with a Dunnett posttest).

**FIGURE 6.**
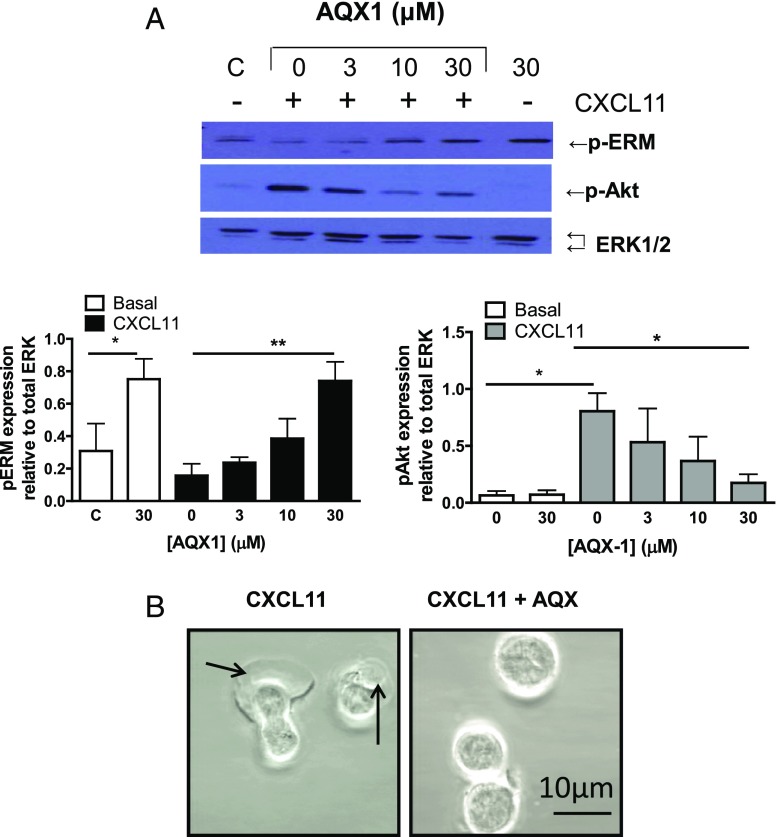
SHIP-1 modulation inhibits polarization and alters the phosphorylation state of ERM proteins in T lymphocytes. (**A**) Previously activated T lymphocytes were incubated either with vehicle control (labeled C) or AQX1 (3–30 μM) for 30 min and then stimulated with CXCL11 (10 nM) as indicated for 5 min. Levels of phosphorylated ERM, phosphorylated Akt, and total ERK were assessed using immunoblotting. The upper panel is a representative Western blot from a single experiment. The right panel shows the mean ± SEM of phosphorylated ERM relative to total ERK, and the left panel shows the mean ± SEM of phosphorylated Akt relative to total ERK from three independent experiments. *p < 0.05, **p < 0.01 (one-way ANOVA with a Dunnett posttest). (**B**) Previously activated T cells were treated with AQX1 (30 μM) and allowed to equilibrate upon poly-l-lysine (0.1 mg/ml)–coated ibidi μ-slides. Cells were stimulated with CXCL11 (100 nM) and lamellipodia extension observed using the Zeiss LSM 510 META microscope.

Similar to the activation of SHIP-1 by H_2_O_2_ and the chemotactic inhibition of T cells toward CXCL11 after treatment with H_2_O_2_, the allosteric SHIP-1 activator AQX1 also significantly impeded previously activated T lymphocyte migration to the same chemokine on a Neuro Probe assay (Fig. 5A). The ability of AQX1 to activate SHIP-1 was verified by direct application of AQX1 on rSHIP-1 (Supplemental Fig. 1B). Migration of T cells in the presence of AQX1 was also assessed using the ibidi μ-slide chemotaxis assay that allows tracking of individual cells across a fibronectin-coated surface (Fig. 5B–D). Pharmacological activation of SHIP-1 was found to abrogate CXCL11-mediated migration as measured by accumulated distance and velocity. Using the ibidi assay, we did not observe a reduction in basal migration in the presence of AQX1. However, this assay has negligible basal migration compared with the Neuro Probe assay. Although pharmacological activation of SHIP-1 had no effect on CXCR3 expression (data not shown), consistent with the impaired migration ability, AQX1-mediated SHIP-1 activation was found to reduce CXCR3/CXCL11-induced phosphorylation of Akt (Fig. 6A).

**FIGURE 7.**
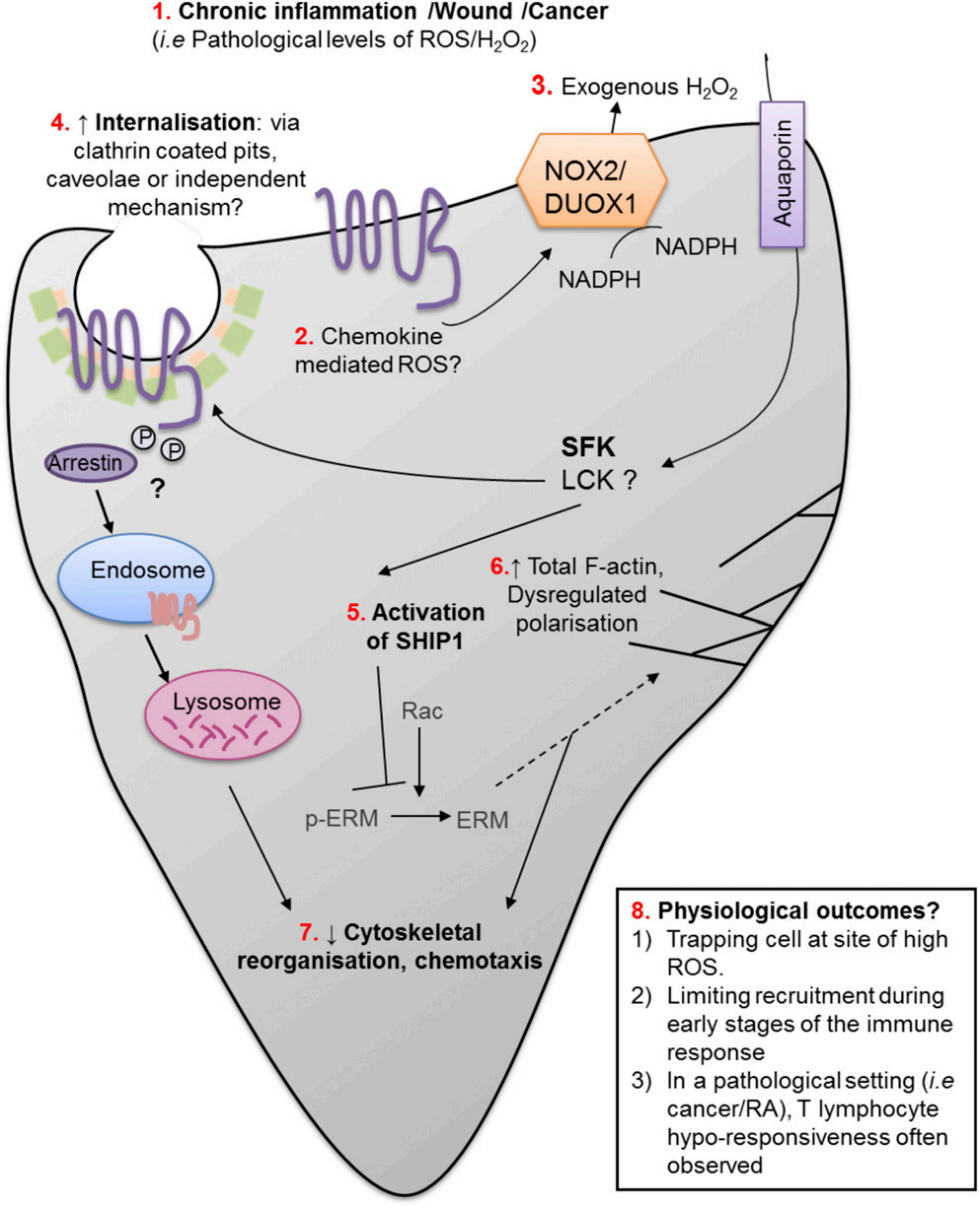
Model of ROS-induced suppression of T lymphocyte migration. (1) Several pathologies result in accumulation of high levels of ROS, including wounds, chronic infection, and cancer. (2) Chemokine stimulation also produces exogenous H_2_O_2_ through NOX2/DUOX enzymes, which are known to be expressed on T lymphocytes. (3) Signaling concentrations of exogenous H_2_O_2_ enters the cell through aquaporin channels and can manipulate proteins in the cytosol. (4) H_2_O_2_ signals through SFKs to internalize chemokine receptor CXCR3; internalization could occur through arrestin recruitment/clathrin-coated pits, caveolae, or an independent mechanism. Once internalized, CXCR3 is degraded in the endosome. (5) H_2_O_2_ also signals through SFKs to phosphorylate and activate SHIP-1. SHIP-1 activation inhibits the ability of CXCL11 to dephosphorylate cytoskeletal proteins ERM, and (6) inhibits actin polarization. H_2_O_2_ also increases total F-actin. (7) Collectively, chemokine internalization, SHIP-1 activation, and actin regulation reduce cytoskeletal reorganization and cell chemotaxis. (8) Proposed outcomes of migration deficiency in T lymphocytes.

Additionally, SHIP-1 activation prevented lamellipodia extension (Fig. 6B). Phosphorylation of ERM proteins, which link the actin cytoskeleton to the cell surface membrane, have previously been implicated in cell migration and adhesion (36). In T lymphocytes, the phosphorylation of ERM proteins is rapidly reduced by chemokine stimulation (37). Additionally, silencing of SHIP-1 expression in T lymphocytes was found to cause the dephosphorylation of ERM proteins (31). Pharmacological SHIP-1 activation by AQX1 treatment enhanced the phosphorylation of ERM proteins in previously activated T lymphocytes under unstimulated conditions, whereas exposure to chemokine CXCL11 (Fig. 6A) caused a dephosphorylation of ERM proteins. However, activation of SHIP-1 with AQX1 prevented CXCL11-mediated ERM dephosphorylation (Fig. 6A). We have provided a model of how we hypothesize ROS suppresses T lymphocyte migration (Fig. 7).

## Discussion

In this study we have shown that H_2_O_2_ pretreatment induced a selective and robust inhibition of T cell chemotactic migration toward CXCL11 but not toward CXCL12 or CXCL10. This implies that H_2_O_2_ has a precise signaling effect on the CXCR3 receptor. We addressed whether H_2_O_2_ treatment could alter the ability of T lymphocytes to adhere, as the adhesion of lymphocytes to components of the extracellular matrix and other cells is critical for successful cell migration, extravasation, and the formation of immunological synapses (38). It has also been previously reported that H_2_O_2_ can alter β_2_ integrin CD11b/CD18 activation and enhance neutrophil adhesion (39). Furthermore, H_2_O_2_ has been described to induce VCAM-1 expression in B cells through extracellular Ca^2+^ influx (40) and enhance leukocyte adhesion to endothelial cells via NF-κβ–dependent gene regulation of VCAM-1 (41). Our experiments (data not shown) revealed that H_2_O_2_ had no effect on either basal or TCR-stimulated adhesion to fibronectin or on expression of the integrin receptors LFA-1 (CD11a expression) and α_4_β_1_ (CD49d expression) in SEB-activated T lymphocytes. Defective adhesion cannot therefore explain the inhibitory effect of H_2_O_2_ on CXCL11-induced migration.

Instead, our results show that H_2_O_2_ has an important role in actin regulation within activated T lymphocytes. H_2_O_2_ significantly increased the level of F-actin and inhibited actin polarization to CXCL11 in SEB-activated T lymphocytes. This effect on the actin cytoskeleton is likely to be one mechanism underlying the ability of high concentrations of H_2_O_2_ to inhibit migration and is consistent with previous studies showing that preventing H_2_O_2_ uptake impairs actin polymerization in murine T lymphocytes (1), whereas oxidative stress enhances actin polymerization and impairs actin polarization to chemokine stimulation in human naive T lymphocytes (42).

To further dissect the mechanism underlying the H_2_O_2_-induced migratory defect, we evaluated the effect of H_2_O_2_ on the surface expression of CXCR3, the cognate receptor of CXCL11. Chemokine receptors, such as CXCR3, undergo internalization and recycling to regulate their signaling. Receptor internalization decreases the amount of available receptor on the cell surface, attenuating receptor-mediated signaling and migration (43, 44). The H_2_O_2_ treatment we performed was limited to 30 min so that changes in surface expression were likely to affect the internalization of the receptor and not the overall expression of the receptor through gene transcription. Consistent with the migratory defect, nontoxic concentrations of H_2_O_2_ significantly decreased the cell surface expression of CXCR3 in our model. Moreover, SFK inhibition completely attenuated this effect. H_2_O_2_ inactivates the phosphatase PTEN (14, 15) and therefore has been presumed to also inactivate the lipid phosphatase SHIP-1, which acts by dephosphorylating the membrane-bound PtdIns(3,4,5)P_3_, generated by PI3K, and has thus been described as a negative regulator of immune receptor, cytokine, and growth factor receptor signaling. Furthermore, SHIP-1 can interact with a large number of proteins via its SH2- and NPXY-containing domains, thereby influencing numerous signaling pathways. We found that low concentrations of H_2_O_2_ induced phosphorylation and enhanced the catalytic activity of cellular SHIP-1 through SFK-dependent signaling. We have shown that SHIP-1 activation severely impedes the migration of activated T lymphocytes and that SHIP-1 activation enhances the phosphorylation of ERM proteins. Although we have not definitively shown that H_2_O_2_ can induce the phosphorylation of ERM in T lymphocytes, it has previously been shown in other cells to be induced by H_2_O_2_ (45), and we therefore suggest that SHIP-1 activation offers one potential mechanism by which H_2_O_2_ impairs T cell migration.

The downregulation of CXCR3 and the enhanced activity of SHIP-1 triggered by H_2_O_2_ could operate in concert to inhibit CXCL11-dependent T cell migration. Because we have shown that both of these effects are dependent on SFK activity, it now becomes important to identify which kinase mediates these responses. The SFK member Lyn has been indicated as a redox sensor involved in early neutrophil recruitment to H_2_O_2_ at wounds. Mutation of a single conserved cysteine residue to alanine at position Cys^466^ abolished the ability of Lyn to be oxidized by H_2_O_2_. This mutation resulted in an inability of the cell to detect and migrate toward an H_2_O_2_ gradient (8). Lck and Fyn are expressed in T lymphocytes and contain the critical cysteine 466 residue, and Lck has been reported to have redox sensitivity (46). Further work is needed to address the precise role of Lck and Fyn in regulating T cell migration downstream of ROS.

There is growing evidence that ROS are not merely an unwanted byproduct of aerobic respiration that cause unwanted damage. Instead, they are critical signaling molecules that are required for coordinating crucial physiological processes. The unconventional concentration-dependent effect with increasing concentrations of H_2_O_2_ likely reflects that H_2_O_2_ is known to have many diverse effects on signaling proteins and, hence, oxidation of different additional proteins could occur at distinct concentrations. We have shown that H_2_O_2_ has significant functional and signaling responses at low physiological concentrations, which have no effect on cell viability.

Previous studies have shown that ROS can influence the migration of innate immune cells in many systems, and in this study we identify a novel effect of ROS on human T cell migration. We propose that such inhibition could dampen the recruitment of adaptive immune cells that are required later in the wound repair process. In contrast, exposure of T cells to low levels of ROS could restrict them to inflamed sites facilitating the resolution of inflammation, whereas in a pathological setting, high ROS levels could cause widespread suppression of T lymphocyte migration and aid cancer cell survival or prolong chronic infection (Fig. 7).

Importantly, the timing and subcellular localization of ROS generation are likely of greater influence in T cell responses than overall redox balance. Interestingly, controlled clinical trials have failed to show a consistent benefit of antioxidants in disease settings (47–49). This supports our observation that oxidants are not solely toxic to the human body and that ROS can be both protective and deleterious depending on concentration and physiological setting.

## Supplementary Material

Data Supplement
