# Supplementary material for: Hydrogen Peroxide Triggers a Dual Signaling Axis To Selectively Suppress Activated Human T Lymphocyte Migration
Source: J Immunol. 2017 Mar 31;198(9):3679–89. doi: 10.4049/jimmunol.1600868 (PMC5392728; doi:10.4049/jimmunol.1600868)
Supplement: Data Supplement [file JI_1600868.zip › JI_1600868_Supplemental_Figures_1.pdf]

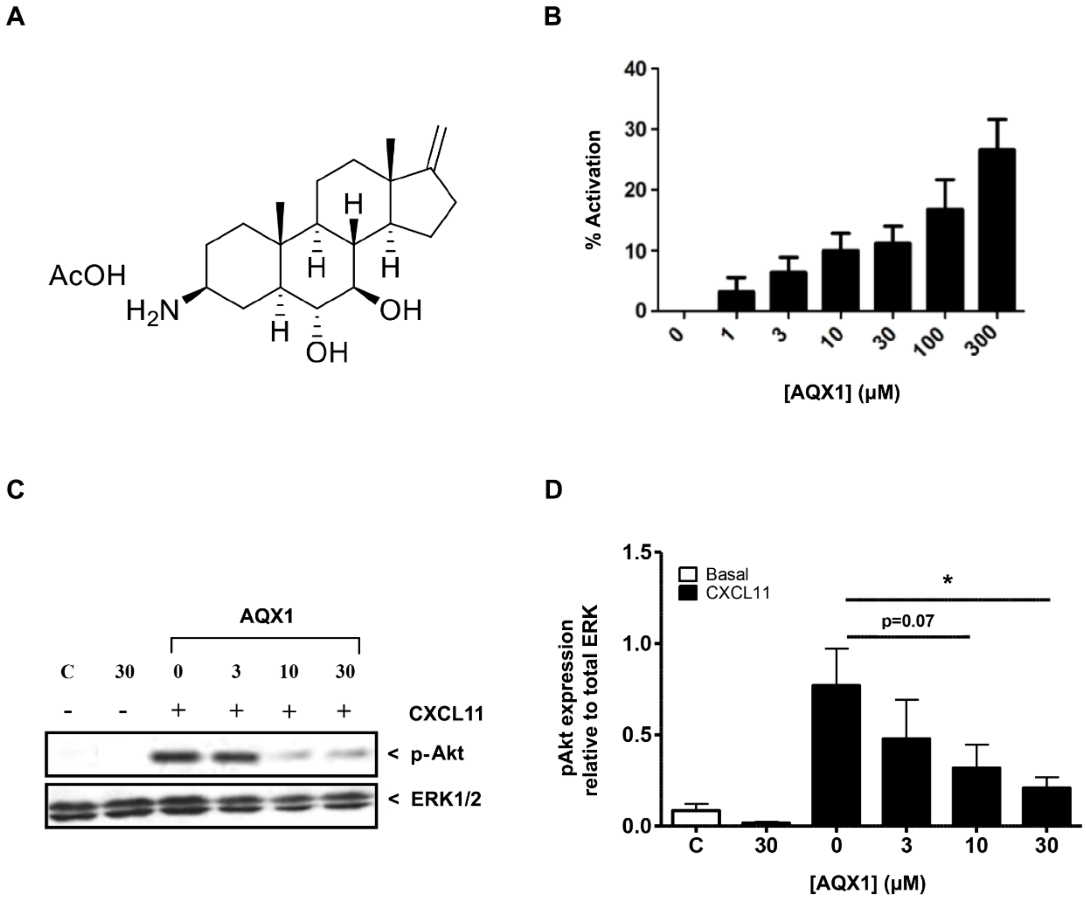

Supplementary Figure 1: (A) Chemical Structure of AQX1. **(B)** To verify the activity of AQX1 on SHIP-1, recombinant full length SHIP-1 (1 µg/µl) was treated with stated concentrations of AQX1 for 15 min then 100 µM IP4 was added for 30 min at 37°C. Activation of T cells was determined using the malachite green assay as described in Material and Methods. Previously activated T cells were treated with either vehicle control (labelled C) or stated concentrations of AQX1 for 30 min and then stimulated with CXCL11 (10 nM) for 5 min. **(C)** Cells were then lysed and level of phosphorylated Akt (serine 473) and total ERK levels determined by immunoblotting. **(D)** The blot was quantified by densitometry using ImageJ software. Data is presented as a representative blot and bar chart of the mean ± SEM of three independent experiments. Significance is represented by *$p<0.05$ (one way ANOVA with Dunnett's post-test).

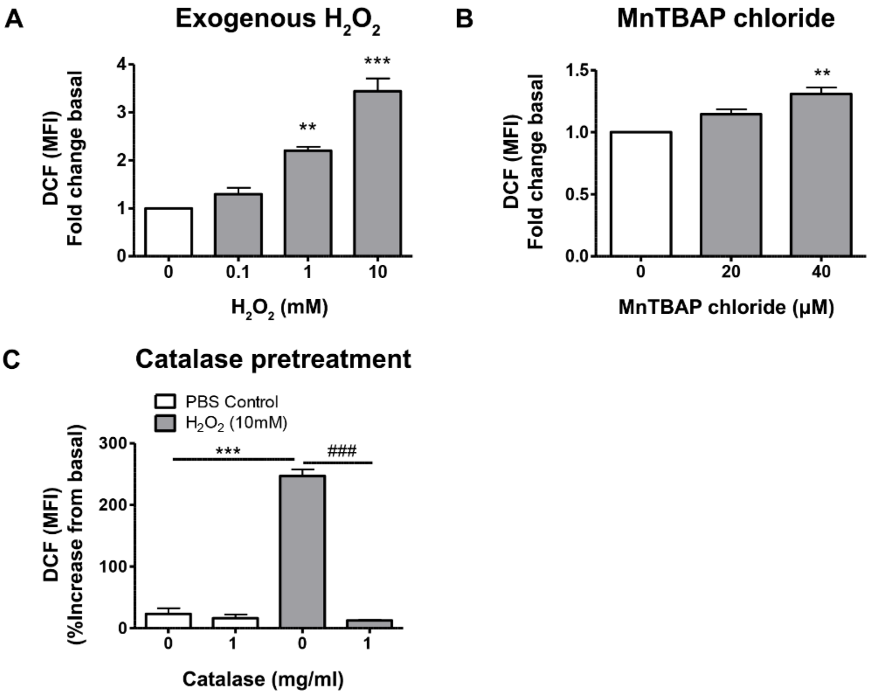

Supplementary Figure 2: Intracellular ROS is increased by H_2O_2/ MnTBAP chloride and reduced by catalase in SEB-activated T lymphocytes. SEB-activated T lymphocytes were loaded with 1µM DCFDA for 30 min. Cells were washed twice in HBSS, resuspended at 1 million cells per ml and treated with stated concentrations of either **(A)** H_2O_2, **(B)** MnTBAP chloride, **(C)** Catalase. For **(A & B)** ROS generation was measured after 30 min using a plate reader and normalised to fold change from basal. For **(C)**, ROS generation was measured using flow cytometry. **(C)** Cells were pre-treated either with PBS control or catalase for 30 min and basal fluorescence reading taken. Then cells were treated with 10mM H_2O_2 for 5 min before fluorescence was measured. Data are the mean ± SEM of three independent experiments. Statistical significance was determined by a one-way ANOVA with Dunnett's post-test where $^{**}p<0.01$ or $^{***}p<0.001$ as compared to control or $^{###}p<0.001$ as compared to H_2O_2 alone.

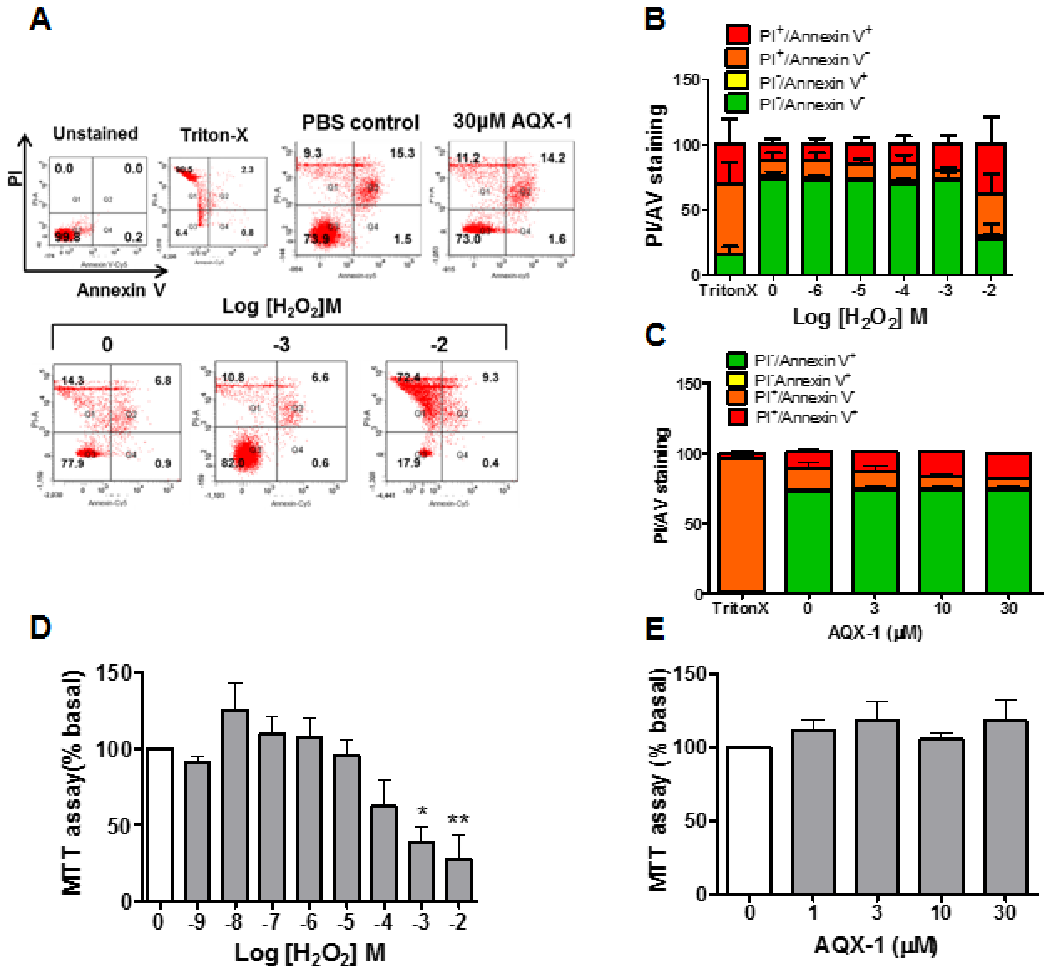

Supplementary Figure 3. Cell viability of SEB activated T lymphocytes treated with H_2O_2 and AQX-1. (A) SEB activated T lymphocytes were treated with increasing concentrations of either H_2O_2 or SHIP-1 activator AQX-1 for 3 hours. Cell viability was measured by PI/Annexin V staining. Representative dual PI/Annexin V stained plots are given. The graph shows **(B)** H_2O_2 and **(C)** AQX-1 the percentage of cells which were PI^- /Annexin V^- in green, PI^- /Annexin V^+ (yellow), PI^+ /Annexin V^- (orange), and PI^+ /Annexin V^+ (Red). Cell viability to increasing concentrations of **(D)** H_2O_2 and **(E)** AQX-1 was also assessed by the MTT assay. Data are mean ± SEM from three independent donors.

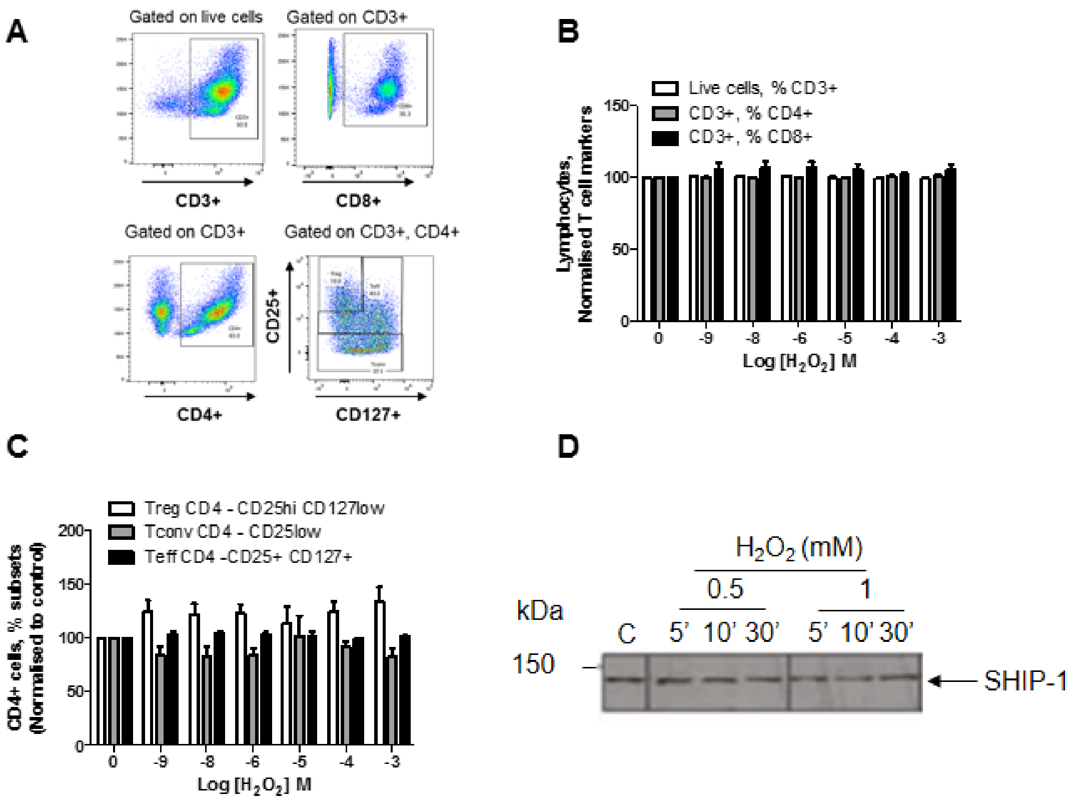

Supplementary Figure 4. H_2O_2 does not significantly alter T lymphocyte subsets (A) SEB activated T lymphocyte were treated with increasing concentrations of H_2O_2 for 30 min. Surface expression of CD3 PerCP , CD4 AF700, CD8 PECy7, CD127 PECy5 and CD25 BV421, in addition to live/dead Zombie yellow, was used to identify the effect of H_2O_2 on T cell subsets. The representative image indicate the gating strategy for all samples. **(B)** Graph showing the effect of H_2O_2 on CD3+, % live cells, or CD4+, % CD3+ and CD8+, % CD3. (C) Graph showing the effect of H_2O_2 on CD4+ subsets. **(D)** A representative Western Blot show the effect of H_2O_2 upon total SHIP-1 protein.
